# Imaging of Fibrosis in Chronic Pancreatitis

**DOI:** 10.3389/fphys.2021.800516

**Published:** 2022-01-10

**Authors:** Yasunobu Yamashita, Reiko Ashida, Masayuki Kitano

**Affiliations:** Second Department of Internal Medicine, Wakayama Medical University, Wakayama, Japan

**Keywords:** chronic pancreatitis, fibrosis, elastography, EUS, ERCP, MRI, CT, imaging

## Abstract

Chronic pancreatitis (CP) describes long-standing inflammation of the pancreas, which leads to irreversible and progressive inflammation of the pancreas with fibrosis. CP also leads to abdominal pain, malnutrition, and permanent impairment of exocrine/endocrine functions. However, it is difficult to assess CP pathologically, and imaging modalities therefore play an important role in the diagnosis and assessment of CP. There are four modalities typically used to assess CP. Pancreatic duct features are assessed with magnetic resonance cholangiopancreatography (MRCP) and endoscopic retrograde cholangiopancreatography (ERCP). However, ERCP is a rather invasive diagnostic modality for CP, and can result in adverse events such as post-ERCP pancreatitis. Computed tomography (CT) is often the most appropriate initial imaging modality for patients with suspected CP, and has high diagnostic specificity. However, CT findings typically only appear in advanced stages of CP, and it is difficult to detect early CP. Endoscopic ultrasonography (EUS) provides superior spatial resolution compared with other imaging modalities such as CT and magnetic resonance imaging (MRI), and is considered the most reliable and efficient diagnostic modality for pancreatic diseases. The EUS-based Rosemont classification plays an important role in diagnosing CP in clinical practice. Evaluation of tissue stiffness can be another option to assess the diagnosis and progression of CP, and MRI and EUS can be used to assess CP not only with imaging, but also with elasticity measurement. MR and EUS elastography are expected to provide new alternative diagnostic tools for assessment of fibrosis in CP, which is difficult to evaluate pathologically.

## Introduction

Fibrosis is seen in a wide variety of benign and malignant diseases of the digestive system. According to a systematic review by Xiao et al. (2016), the global pooled incidence rate of chronic pancreatitis (CP) is 10 per 100,000 person-years (95% confidence interval [CI]: 8–12). Nationwide epidemiological surveys in Japan have demonstrated an increasing prevalence of CP, from 28.5/100,000 in 1994 to 44.5/100,000 in 2016 (Lin et al., 2000; Masamune et al., 2020). Therefore, CP is currently considered one of the most important healthcare problems. CP is characterized as chronic inflammation of the pancreas with fibrosis and permanent impairment of exocrine and endocrine functions, resulting in irreversible structural damage. Moreover, CP is a risk factor for developing pancreatic cancer. In terms of pancreatic fibrosis, a two-hit theory has been proposed. The first hit is acute pancreatitis, which causes injury to the pancreas. The second hit is an abnormal inflammatory response to injury. This causes sustained activation of pancreatic profibrotic cells including stellate cells. These responses result in collagen deposition and fibrosis, and finally lead to CP (Barry, 2018). The end stage of CP presents multiple complications such as pain, pancreatic insufficiency, metabolic bone disease, and pancreatic cancer.

However, it is difficult to perform histological evaluations of CP, and only a few patients undergo surgical resection. Moreover, DeWitt et al. (2005) reported that endoscopic ultrasonography-guided Trucut biopsy (EUS-TCB) is inadequate for evaluating CP grade because of histopathological heterogeneity. Although fibrosis of the pancreas is assessed according to endocrine and exocrine dysfunction in clinical practice, physiological markers such as these cannot accurately detect fibrotic change. Therefore, if imaging modalities are able to evaluate the elasticity of tissue and internal stricture, they can be used as alternative diagnostic tools with performance close to that of pathology.

Parenchyma and pancreatic duct features are typically assessed in the imaging diagnosis of CP. Magnetic resonance cholangiopancreatography (MRCP) and endoscopic retrograde cholangiopancreatography (ERCP) are mainly used to examine pancreatic duct features, whereas EUS is able to assess both parenchymal and pancreatic duct features. Computed tomography (CT) is mainly used for the diagnosis of CP according to parenchymal features. Imaging modalities can also be used to evaluate tissue stiffness, which can be used to diagnose and assess the progression of CP. We here provide an overview of imaging modalities for the diagnosis of CP with fibrosis, discussing the diagnostic abilities of each tool for determining progression.

## Imaging Modalities

### Endoscopic Retrograde Cholangiopancreatography

Endoscopic retrograde cholangiopancreatography has a superior spatial resolution and ability to depict side branch abnormalities. Therefore, ERCP was previously the gold standard for the diagnosis of CP. Moreover, it is possible to perform therapeutic interventions such as dilation for pancreatic duct stenosis, stone extraction, and stenting of the pancreatic duct, as well as cytological evaluation with pancreatic juice for pancreatic cancer concomitant with CP. ERCP also has the advantage of detecting subtle duct lesions such as pancreatic divisum. The diameter of the normal main pancreatic duct (MPD) depends on the sites of the pancreas (3–4 mm in the head, 2–3 mm in the body and 1–2 mm in the tail region). Multiple side branches join the MPD at right angles in an alternating fashion. ERCP allows detection of pancreatic duct abnormalities, including ductal dilation, stricture, abnormal side branching, communicating pseudocyst, pancreatic duct stone, and pancreatic duct leakage. The features of early CP on ERCP are irregularity and dilatation of side branches. Intraductal calculi can be seen as filling defects. As CP progresses, these changes can become more severe, together with dilatation, loss of normal tapering and irregularity of the MPD (Shanbhogue et al., 2009). ERCP is highly effective at visualizing these ductal findings, with sensitivity of 71–93% and specificity of 89–100% (Christodoulou and Tsianos, 2010). According to the Japan Pancreas Society (JPS) 2019, the imaging findings of early CP on ERCP are irregular dilatation of more than three side branches. However, ERCP is limited because it does not allow evaluation of the pancreatic parenchyma. Moreover, ERCP is the most invasive of the diagnostic modalities for CP, with the possibility for adverse events such as post-ERCP pancreatitis. Therefore, ERCP should be performed when the diagnosis of CP is still unclear after non-invasive CT, MRI, and less-invasive EUS have been performed in patients with suspected CP.

### Computed Tomography

Computed tomography is the most common imaging modality used for the initial diagnosis of CP. Axial images should be reconstructed preferably at a thickness of less than 2.5 mm. CT should include non-enhancement to identify calcifications and enhancement to detect pseudoaneurysms, pseudocysts, and focal lesions including duodenal and biliary stenosis, and for pancreatic parenchymal and duct evaluation. For normal pancreas, CT detects a homogeneous structure with smooth lobulated borders. The typical imaging findings of CP on CT are dilation of the MPD, pancreatic calcification, pancreatic atrophic change, and pancreatic pseudocyst. Dilation of the MPD, pancreatic calcification, pancreatic atrophic change, and pancreatic pseudocyst were detected in 68, 50, 54, and 30% of patients with CP, respectively (Anaizi et al., 2017). The calcification can vary in size and morphology and the degree of calcification is directly proportional to the duration of the disease. The pancreatic head is the commonest location of parenchymal calcifications (Lankisch et al., 1986; Lesniak et al., 2002). Although diagnosis of early CP is not reliable, CT should be performed for all patients to exclude the possibility of a mass or complication of CP.

### Magnetic Resonance Imaging

#### Conventional Magnetic Resonance Imaging

Magnetic resonance imaging allows detection of the morphological presentations of pancreatic fibrotic change. A normal pancreas appears hyperintense on T1-weighted sequences with or without fat saturation. The degree of its intensity is the highest among abdominal structures with the exception of fatty liver (Winston et al., 1995). The fibrotic replacement of parenchyma, responsible for the reduced protein content, can be detected as parenchymal signal intensity change on T1-weighted imaging. In this process, the pancreatic parenchyma loses its normal high-signal-intensity appearance related to a high protein content.

On the other hand, pancreatic duct abnormalities consisting of the appearance of side branches in the initial stages of disease and more severe irregularity of the MPD in advanced stages are well depicted on MRCP images. The ductal changes are better visualized on MRCP than on CT. Moreover, MRCP has replaced ERCP for the diagnostic imaging of biliary and pancreatic ducts. However, the side branches are not clearly visualized (Takehara et al., 1994). Secretin increases the absolute volume of intraductal free water and fills the collapsed branches with fluid because secretin stimulates fluid secretion in the ductal system, and increases the tonus of the sphincter of Oddi during the first 5 min, hindering the release of fluid through the papilla of Vater (Cappeliez et al., 2000). Addition of secretin enhancement can improve visualization of abnormalities of the pancreatic duct and its branches, which may not be seen on routine MRCP (Cappeliez et al., 2000). MRCP detects stricture of the MPD, irregular contour of the MPD, dilated side branches, and filling defects due to pancreatic stones and protein plugs in CP. MRCP also facilitates the diagnosis of complications of CP such as biliary strictures and pseudocysts. The presence of two or more features predicts CP with 65% sensitivity, 89% specificity, and 68% accuracy (Trikudanathan et al., 2015). These findings also predict fibrosis with 88% sensitivity and 78% specificity (Trikudanathan et al., 2015).

Diffusion-weighted imaging (DWI) is also performed to evaluate CP using apparent diffusion coefficient (ADC) values. Diffusion is restricted because the exocrine reserve of the pancreas leads to decreased water diffusion and fibrosis. Therefore, ADC values are lower in patients with CP than in normal patients. ADC values can potentially be used as an indicator of fibrosis and of its extent in patients with CP. Furthermore, a combination of DWI and secretin-enhanced MRCP (S-MRCP) increases ADC values. In normal patients, ADC values are expected to increase during the early part of S-MRCP studies. On the other hand, this peak in ADC values delayed or does not occur in patients with CP (Procacci et al., 1997).

#### Magnetic Resonance Elastography

Magnetic resonance elastography is another option, allowing the diagnosis of CP according to elasticity measurement. MR elastography requires five components: (1) a driver system to continuously generate oscillatory mechanical waves at a fixed frequency; (2) a phase-contrast multiphase pulse sequence with motion-encoding gradients that are synchronized to the mechanical waves; (3) processing of phase-sensitive MR images to depict wave amplitudes (shear-wave displacement images or, simply, wave images); (4) further post-processing to generate elastograms (using an inversion algorithm); and (5) analysis of the elastograms.

The estimation of pancreatic stiffness in CP patients with MR elastography was reported in two studies. An et al. (2016) reported that patients with CP had significantly higher mean stiffness values than healthy controls (1.53 vs. 1.11 kPa). Wang et al. (2018) reported that overall pancreatic stiffness significantly differed between healthy controls (mean: 1.21 kPa), patients with a mild degree of CP (mean: 1.50 kPa), and patients with a moderate/severe degree of CP (mean: 1.90 kPa). MR elastography showed a good performance for assessment of CP severity. These reports showed significantly higher stiffness levels for the patients with CP (An et al., 2016; Wang et al., 2018).

### Endoscopic Ultrasonography

#### Conventional Endoscopic Ultrasonography

As mentioned above, CT findings tend to appear in advanced CP disease. Although advanced CP is irreversible, Ito et al. (2015) proposed that early CP can be considered a reversible pathological condition. Therefore, diagnosing CP early is clinically important to prevent pancreatic fibrosis, progression, and complications. However, diagnosis of early CP can be difficult because of a lack of sensitive serum-based functional biomarkers. EUS provides superior spatial resolution to CT, and is considered the most reliable and efficient diagnostic modality for pancreatic diseases. Therefore, EUS has emerged as an important imaging modality for the detection of early morphologic changes in CP, facilitating detection of mild parenchymal and ductal changes not visible on CT (Morris-Stiff et al., 2009). To meet the need for clear guidelines for EUS, the JPS proposed the JPS criteria (JPSC) and a new concept called early CP in 2009 (Shimosegawa et al., 2010). These guidelines described EUS as a diagnostic modality for early CP. Nowadays, EUS-based methods are used for diagnosing CP worldwide. The utility of EUS for the diagnosis of CP was first reported in 1988, and several criteria were proposed. It was, however, limited by a lack of consideration of the disease stage displaying specific CP characteristics. Faced with this situation, the Rosemont classification (RC) was introduced by a group of EUS experts at an international conference (Catalano et al., 2009). Although the RC is widely used in its current status, it does not have high inter-observation agreement, with a Fleiss’ kappa (K) statistic of 0.65 (95% CI, 0.52–0.77) for qualitative assessment (Stevens et al., 2010).

**FIGURE 1:**
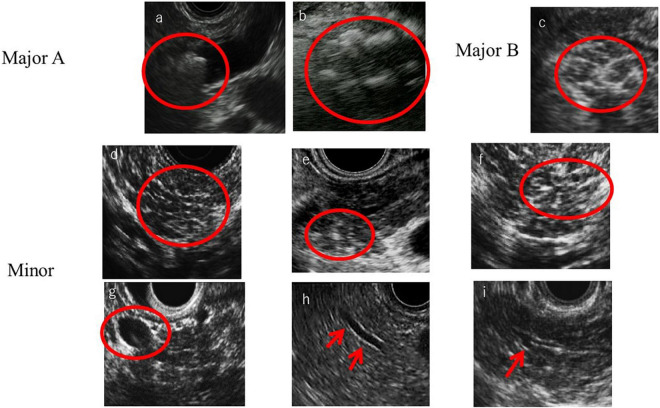
Rosemont criteria items. **(a)** MPD calculi, **(b)** Hyperechoic foci with shadowing, **(c)** Lobularity with honeycombing, **(d)** Lobularity without honeycombing, **(e)** Hyperechoic foci without shadowing, **(f)** Strand, **(g)** Cyst, **(h)** Hyperechoic main pancreatic duct margin, and **(i)** Dilated side branches.

In the RC, the ductal and parenchymal EUS findings are divided into major A criteria, major B criteria, and minor criteria. Major A criteria consist of hyperechoic foci with shadowing and MPD calculi. This feature is defined as the presence of echogenic structures ≥ 2 mm in length and width that produce a shadow. Major B criteria consist of lobularity with honeycombing. Loburality is defined endosonographically as a well-circumscribed structure measuring ≥ 5 mm with rims that are hyperechoic relative to the echogenicity of its central area. When at least three of the lobules are contiguous, the features are considered Major B criteria. Minor criteria consist of hyperechoic strands, hyperechoic foci without shadowing, lobularity without honeycombing, cyst, MPD dilation, irregular MPD contour, dilated side branch, and hyperechoic MPD margin (Figure 1). The RC classifies EUS findings as “consistent with CP,” “suggestive of CP,” “indeterminate for CP,” or “normal.” (Catalano et al., 2009). Zubarik and Ganguly (2011) reported that individual RC criteria of hyperechoic foci with shadowing, lobularity, and stranding were associated with improvement of pain after pancreatic enzyme supplement therapy. The sensitivity and specificity of exocrine dysfunction for an EUS diagnosis of CP using the RC were 23.4 and 78.6%, respectively. However, the diagnosis of exocrine dysfunction showed a poor correlation with RC criteria for diagnosis of CP (Zubarik and Ganguly, 2011). Regarding the associations between the RC classifications and the histology of pancreatic fibrosis, the percentages of pathological findings (fibrosis change) of CP were 96.2% in the classification “suggestive of CP,” 80% in “indeterminate for CP,” and 55.5% in “normal” (Trikudanathan et al., 2017). Therefore, when RC indicates normal pancreas but patients have clinical signs of suspected CP such as abdominal pain or abnormal pancreatic enzyme level, the RC cannot rule out CP. Moreover, in 21% of patients in whom initial EUS imaging with the RC failed to diagnose CP, CP then developed during a mean follow-up of 7 years (Monachese et al., 2021).

In 2009, the JPS first proposed diagnostic criteria for early CP, aiming to improve the long-term prognosis of patients with CP through early diagnosis and therapeutic intervention (Shimosegawa et al., 2010). A revised edition of the JPSC was published in 2019. In addition to imaging findings of early CP on EUS, MRCP, or ERCP, the diagnosis of early CP according to JPSC 2019 requires more than three of the following clinical signs: repeated upper abdominal or back pain; abnormal pancreatic enzyme levels in serum or urine; abnormal pancreatic exocrine function; continuous heavy drinking of alcohol equivalent to or more than 60 g/day of pure ethanol; or mutation in a pancreatitis-associated gene and an imaging finding of early CP on EUS, MRCP, or ERCP. In particular, EUS plays an important role in the detection of early CP in clinical practice. Imaging findings of early CP on EUS consist of four items: (1) hyperechoic foci without shadowing or strands; (2) lobularity; (3) hyperechoic MPD margins; and (4) dilated side branches. Early CP is diagnosed on EUS according to two or more of these four findings, including hyperechoic foci without shadowing, strands or loburality. By using these criteria, it may be possible to diagnose early CP in cases diagnosed as normal by the RC. Therefore, JPSC 2019 is considered better for the EUS assessment of pancreatic fibrosis in CP (Masamune et al., 2020).

#### Endoscopic Ultrasonography Elastography

Recently, EUS elastography, which measures tissue hardness, has become another option for the diagnosis of CP. EUS elastography is a novel diagnostic method based on the measurement of tissue elasticity, and evaluation of tissue stiffness can be used to assess fibrosis of the pancreas in CP. EUS elastography can be classified into two categories on the basis of the different mechanical properties evaluated: strain elastography and shear-wave elastography (SWE).

##### Endoscopic Ultrasonography Strain Elastography

**FIGURE 2:**
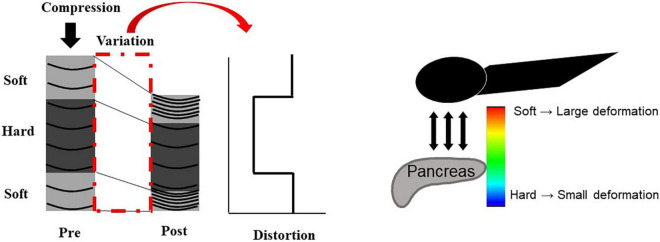
Illustration of EUS strain elastography. The strain created by compression of the target tissue with the EUS probe or cardiovascular pulsation through the aorta is expressed on ultrasound images. A larger strain indicates softer tissue, whereas a smaller strain reflects harder tissue. Strain is exhibited *via* different colors based on the elasticity of the tissue: red indicates soft tissues, and blue indicates hard tissues.

**FIGURE 3:**
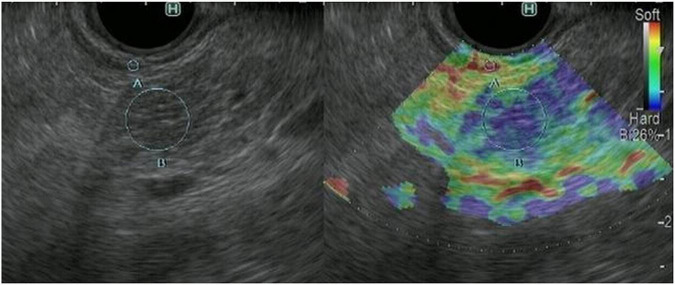
Representative EUS strain elastography images in a patient with chronic pancreatitis. EUS strain elastography measurements in pancreatic parenchyma are shown mainly in blue, indicating hardness.

The principle underlying EUS strain elastography is that the strain created by compression of the target tissue with the EUS probe or cardiovascular pulsation through the aorta is expressed on ultrasound images. A larger strain indicates softer tissue, whereas a smaller strain reflects harder tissue. Strain is exhibited *via* different colors based on the elasticity of the tissue: red indicates soft tissues and blue indicates hard tissues (Figures 2, 3). This evaluation of tissue elasticity is qualitative, although two semi-quantitative measures of tissue stiffness are now possible with the development of second-generation EUS elastography. The strain ratio (SR) is based on a comparison between regions of interest (ROIs) in two tissue areas. The SR is a semi-quantitative parameter because the hardness is expressed as a relative ratio, not as an absolute value. Another method is the strain histogram (SH). The SH represents the mean strain value of the selected area, with the graph produced with SH software representing elasticity values from 0 to 255, with 0 being the hardest and 255 the softest. There are six articles reporting the utility of EUS strain elastography for CP, three of these reporting the use of SR, and three using SH (Machado et al., 2012; Iglesias-Garcia et al., 2013; Itoh et al., 2014; Dominguez-Muñoz et al., 2015; Kim et al., 2017; Kuwahara et al., 2017). Two of the three SR articles reported that EUS elastography is useful for the differentiation between normal pancreas and CP (Iglesias-Garcia et al., 2013; Kim et al., 2017). Moreover, one of the two articles reported that EUS strain elastography values correlated with the number of RC criteria (Iglesias-Garcia et al., 2013). Another report showed that SR EUS elastography values were significantly correlated with exocrine dysfunction (Dominguez-Muñoz et al., 2015). Two of the three SH articles also reported that EUS elastography is useful for the differentiation between normal pancreas and CP (Machado et al., 2012; Kuwahara et al., 2017). Moreover, one of the two articles reported that SH EUS elastography values correlated with the number of CP criteria and the CP stages of the RC (Kuwahara et al., 2017). Another report showed that the degree of fibrosis histologically assessed on surgical specimens significantly correlated with SH elastography values (Itoh et al., 2014).

##### Endoscopic Ultrasonography Shear Wave Elastography

**FIGURE 4:**
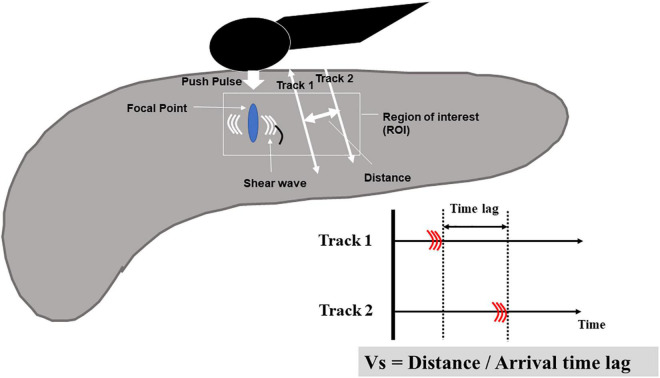
Illustration of EUS shear-wave elastography. Acoustic radiation force (a push pulse) is sent to the focal point of the regions of interest and a shear wave is generated at the edge by this push pulse. The shear wave velocity (distance/arrival time lag [Vs, m/s]) is calculated between two search points with the track pulse.

**FIGURE 5:**
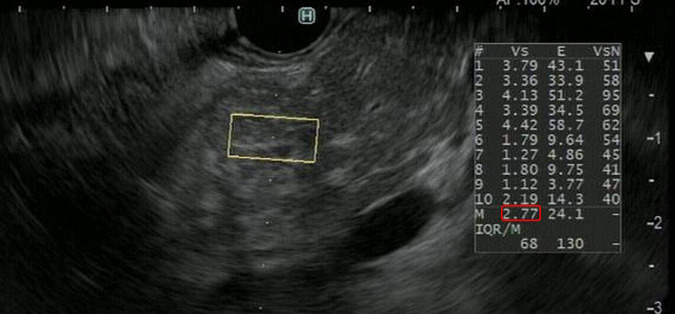
Representative EUS shear wave elastography (EUS-SWE) images in a patient with chronic pancreatitis. EUS-SWE was performed to diagnose chronic pancreatitis. The shear wave velocity (distance/arrival time lag [Vs, m/s]) value of 2.77 (displayed in red square) for the region of interest (yellow square) was higher than the cut-off Vs values of 2.19 and 1.96 for diagnosing chronic pancreatitis.

In the principle underlying EUS-SWE, acoustic radiation force (a push pulse) is sent to the focal point of the ROI and a shear wave is generated at the edge by this push pulse. The shear wave velocity (distance/arrival time lag [Vs, m/s]) is calculated between two search points with the track pulse. If the tissue is hard, the shear wave propagates faster (Figures 4, 5). Although conventional EUS and EUS strain elastography cannot measure absolute values of hardness, EUS-SWE is a more precise modality for diagnosing CP because it can provide absolute values of pancreatic hardness. There are only two reports of the usefulness of EUS-SWE because it is a novel modality in the field of EUS (Yamashita et al., 2020, 2021). For diagnosis of CP, a cut-off Vs value of 2.19 with the RC criteria and 1.96 with JPSC had sensitivity of 100 and 83%, respectively, and specificity of 94 and 100% (Yamashita et al., 2020, 2021; Figure 5). One article reported that EUS-SWE values correlated with the number of RC criteria and the stage of CP according to the RC (Yamashita et al., 2021). Another article reported that EUS-SWE values positively correlated with JPSC and predicted exocrine dysfunction (Yamashita et al., 2021).

## Conclusion

Although CT has high specificity for CP, it is limited in the detection of early CP, for which it has low sensitivity. However, EUS can detect early CP that cannot be detected on CT. In the future, a diagnostic strategy for CP needs to be established by comparing imaging modalities. In particular, elastography with MRI or EUS is expected to become a new diagnostic tool for CP.

## Author Contributions

YY: drafting of the manuscript. RA and MK: critical revision of the manuscript for important intellectual content. MK: final approval of the manuscript. All authors contributed to the article and approved the submitted version.

## Conflict of Interest

MK has received honoraria from Olympus Corporation for speaking lectures at conferences. The remaining authors declare that the research was conducted in the absence of any commercial or financial relationships that could be construed as a potential conflict of interest.

## Publisher’s Note

All claims expressed in this article are solely those of the authors and do not necessarily represent those of their affiliated organizations, or those of the publisher, the editors and the reviewers. Any product that may be evaluated in this article, or claim that may be made by its manufacturer, is not guaranteed or endorsed by the publisher.
